# Changes in Liver Stiffness and Markers of Liver Synthesis and Portal Hypertension following Hepatitis C Virus Eradication in Cirrhotic Individuals

**DOI:** 10.3390/biology11081160

**Published:** 2022-08-02

**Authors:** Angelo Armandi, Chiara Rosso, Giulia Troshina, Nuria Pérez Diaz Del Campo, Chiara Marinoni, Aurora Nicolosi, Gian Paolo Caviglia, Giorgio Maria Saracco, Elisabetta Bugianesi, Alessia Ciancio

**Affiliations:** 1 Department of Medical Sciences, University of Turin, 10126 Turin, Italy; yulia.troshina@unito.it (G.T.); nuria.perezdiazdelcampo@unito.it (N.P.D.D.C.); 20034507@studenti.uniupo.it (C.M.); aurora.nicolosi@unito.it (A.N.); gianpaolo.caviglia@unito.it (G.P.C.); giorgiomaria.saracco@unito.it (G.M.S.); elisabetta.bugianesi@unito.it (E.B.); alessia.ciancio@unito.it (A.C.); 2 Metabolic Liver Disease Research Program, I. Department of Medicine, University Medical Center of the Johannes Gutenberg-University, 55131 Mainz, Germany; 3 Division of Gastroenterology, Città Della Salute e Della Scienza University-Hospital, 10100 Turin, Italy

**Keywords:** hepatitis C virus, direct antiviral agents, hepatic fibrosis, liver stiffness, portal hypertension, cirrhosis, RESIST-HCV

## Abstract

**Simple Summary:**

Liver cirrhosis is a dynamic process that may display improvements when the etiological factor is removed. In this retrospective study of HCV-cured cirrhotic patients, we evaluated changes in liver synthesis, surrogate markers of portal hypertension as well as liver stiffness before starting the antiviral treatment and following successful viral eradication.

**Abstract:**

The advent of direct antiviral agents (DAAs) has radically changed the natural history of hepatitis C virus (HCV) chronic liver disease. Even patients with cirrhosis may display improvements in liver function or features of portal hypertension following viral eradication. The aim of this study was to assess whether a HCV cure would lead to improvements in cirrhotic patients using simple, readily available tools in clinical practice, together with liver stiffness (LS) measurement. This is a retrospective study of cirrhotic patients with cured HCV infection, with or without previous decompensation. Clinical and biochemical parameters as well as LS measurements were collected before antiviral treatment with DAAs and after 6 months following sustained virological response. Hepatic synthesis was assessed by serum albumin levels. Portal hypertension was indirectly assessed by platelet count. Liver function was determined by the CHILD score. A total of 373 cirrhotic patients with successful HCV eradication were retrospectively included. After 6 months of follow-up, a significantly higher proportion of patients showed improved liver function, shifting from the CHILD B/C to CHILD A group, (71.4%, *p* < 0.001). Similarly, LS improved from a median of 19.3 kPa (14.7–27) at the baseline vs. a median of 11.6 (7.7–16.8 kPa) at follow-up (*p* < 0.001). The proportion of patients who showed improved hepatic synthesis was 66.0%, which was statistically different when compared to that of patients who had a worsened condition (0.3%) (*p* < 0.001). Moreover, when classifying the cohort according to the RESIST-HCV score, we found that a significant proportion of patients shifted into the “low risk” group following DAA treatment (52% baseline vs. 45.6% at follow-up, *p* = 0.004). Even in the decompensated patients, LS improved from 1.6 to 2-fold from the baseline. Antiviral treatment is effective in improving indirect signs of hepatic synthesis and portal hypertension. Similarly, the LS values displayed significant improvements, even in decompensated patients.

## 1. Introduction

The advent of direct antiviral agents (DAAs) for a hepatitis C virus (HCV) infection cure has revolutionized the natural history of this chronic liver disease [1]. With more than 98% of treatment success (long-term sustained virological response [SVR], DAAs have allowed for the control of HCV infection worldwide, with the aim of eradicating it within 2030 [2]. 

Liver cirrhosis is an irreversible process that represents the final stage of the natural history of liver disease of any etiology. However, patients with HCV infection undergoing successful antiviral treatment display an improvement in liver function due to the alleviation of HCV-driven intrahepatic inflammation [3]. In fact, the eradication of HCV is associated with significant improvements in liver stiffness (LS), which is among the most accurate non-invasive tools to assess the fibrosis stage [4,5,6]. This is clear even when considering the onset of portal hypertension, which is responsible for overt decompensation [7]. Cirrhotic patients that obtain SVR show improvements in features of portal hypertension, defined by changes in hepatic portal vein gradient (HVPG) [8]. From this perspective, the long-term monitoring of hepatic function in cirrhotic patients is warranted. Despite HVPG being the gold standard to assess portal hypertension, it is an invasive technique, burdened by high costs and limited accessibility. Therefore, non-invasive tools are highly requested in routine clinical activity. Platelet count reduction, for instance, is the earliest sign of portal hypertension occurring in cirrhotic patients. Similarly, the CHILD score is a strongly validated system to assess liver function through clinical and biochemical signs including serum albumin concentration. We conducted a retrospective study of consecutive patients with cirrhosis due to HCV infection undergoing successful eradication therapy, aiming to evaluate whether HCV cure would lead to changes in LS and biochemical markers of liver function and portal hypertension. 

## 2. Materials and Methods

### 2.1. Patients

We retrospectively analyzed data from 2698 consecutive HCV infected patients who received a treatment of DAAs according to the European Association for the Study of the Liver (EASL) guidelines [1]. All patients were enrolled and treated in the outpatient liver clinics of Città della Salute e della Scienza University Hospital, Turin, Italy, from January 2015 to 31 December 2018. 

**Figure 1:**
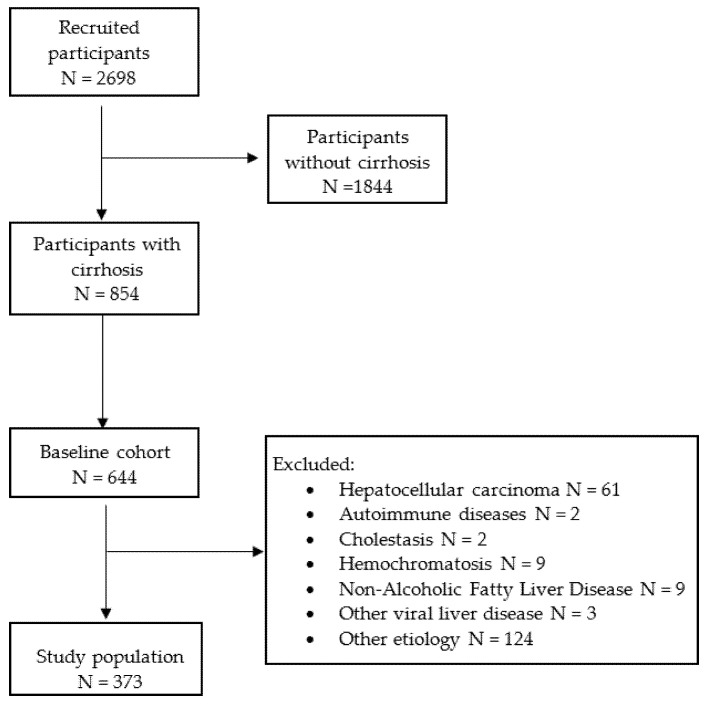
The flow chart of the study.

The inclusion criteria of this study were age >18 years, HCV-RNA positivity by polymerase chain reaction, and the presence of cirrhosis, with or without portal hypertension. Cirrhosis was indirectly diagnosed for patients with previous decompensation. Compensated cirrhosis was defined as the absence of any clinical signs of decompensation (including ascites, esophageal varices bleeding and hepatic encephalopathy), LS >10 kPa (defined as chronic compensated advanced liver disease [cACLD] according to Baveno criteria) [9], or abdominal ultrasound showing irregular morphology of the liver. Portal hypertension was indirectly assessed by low platelet count (<150 × 10^9^/L). Patients were included if they had one follow-up visit at least 6 months following SVR. Cirrhotic patients with unfeasible transient elastography or unreliable measurements were excluded from the analysis. Exclusion criteria were as follows: patients on a waiting list for orthotropic liver transplant (OLT), post-OLT patients, past or current history of hepatocellular carcinoma (HCC), concomitant liver diseases such as haemochromatosis, Wilson’s disease, drug-related liver disease, autoimmune hepatitis, HBsAg carriership, human immunodeficiency virus (HIV) infection, primary biliary cholangitis, and alpha-1-antitrypsin deficiency. Of the total cohort receiving DAA treatment, 644 fulfilled the inclusion criteria. A total of 271 were lost at follow-up. The study population included 373 patients. A flow-chart of the study is reported in Figure 1.

### 2.2. Evaluation of LS and Indirect Signs of Liver Function and Portal Hypertension

All patients underwent LS prior to antiviral therapy. LS was evaluated by transient elastography (Fibroscan) and performed at the fasting condition by an expert operator blinded to the patient information. All measurements were taken with an M probe at 50 Hz between the 6^th^ and the 9^th^ intercostal space. All measurements were considered technically reliable according to the interquartile range (IQR)/median range below 30%. The baseline clinical and biochemical data were collected at the time of the Fibroscan.

For the purpose of the study, the serum albumin levels were considered as an index of hepatic synthesis; values <3.5 g/dL were considered as impaired synthesis [10]. The CHILD score was used to assess the liver function and calculated as described in the literature [11] using the established grades (A, B, and C). Low platelet count was used as a surrogate marker of portal hypertension with a cut-off value of 150 × 10^9^/L as defined by the Baveno criteria [9]. In addition, we combined the albumin levels and platelet count into the RESIST-HCV score, which has been shown to accurately predict the presence of high-risk esophageal varices before DAA treatment, leading to about 30% of endoscopy sparing [12]. In addition, the RESIST-HCV score has shown a robust accuracy to predict high-risk esophageal varices following SVR [13]. The RESIST-HCV score combines the platelet and albumin values according to specific cut-offs: the “low risk” group is defined by albumin levels >3.6 g/dL and platelet count >120 × 10^9^/L.

Follow-up evaluation was performed 6 months following SVR at 24 weeks and included LS, clinical, and biochemical data.

### 2.3. Statistical Analysis

Continuous variables were reported as the median and IQR while categorical variables were reported as the number and percentage. Comparisons between the baseline and follow-up data were performed by the Wilcoxon test for continuous variables or by the McNemar test for categorical variables. For the purpose of the analysis, albumin levels, platelet count, and Child–Pugh score were dichotomized according to the following cut-off: 3.5 g/dL for albumin, 150 × 10^9^/L for platelets, and Child–Pugh score A vs. BC. Statistical analyses were performed using MedCalc^®^ v.18.9.1 (MedCalc Software Ltd., Ostend, Belgium), and a *p* value ≤ 0.05 was considered as statistically significant.

## 3. Results

Baseline characteristics of the study cohort are reported in Table 1. The median age was 64 years and 41.5% of the patients were males. At the baseline, the majority of the cases had CHILD A cirrhosis (94.4%). Portal hypertension and low hepatic synthesis were present in more than 80% of the total cohort. The median baseline LS was 19.3 (14.7–27.0 kPa), while the median platelet count and albumin levels were 119 (86–158) × 10^9^/L and 4.1 (3.7–4.4) g/dL, respectively. The DAA regimens used to treat HCV in this cohort are shown in Supplementary Table S1. Overall, 53.6% of the total were administered Sofosbuvir/Ledipasvir, with or without Ribavirin, followed by Sofosbuvir alone, with or without Ribavirin, in 13.9% of cases.

When classifying the cohort according to the RESIST-HCV score, we found that a significant proportion of patients shifted into the “low risk” group following DAA treatment (52% baseline vs. 45.6% at follow-up, *p* = 0.004).

Follow-up data were collected 6 months following SVR. At follow up, a significantly higher proportion of patients improved liver function, shifting from the CHILD B/C to CHILD A group, while few patients with CHILD A at baseline became CHILD B/C (71.4% vs. 2.3%, *p* < 0.001). Similarly, LS showed an overall improvement at follow-up evaluation: median 19.3 kPa (14.7–27) vs. median 11.6 (7.7–16.8 kPa) at follow-up, *p* < 0.001. 

The variables are shown as the median (IQR) according to its distribution. Categorical variables are presented as absolute (*n*) and relative frequencies (%). Abbreviations: ALT—alanine aminotransferase; AST—aspartate aminotransferase; BMI—body mass index; GGT—gamma-glutamyl transferase.

When using the aforementioned cut-offs (Table 2), we found that the proportion of patients who improved in the indirect signs of portal hypertension was 19.4%, whereas the proportion of patients who had a worsened condition was 17.5% (*p* = 0.853). Conversely, the proportion of patients who had improved hepatic synthesis was 66.0%, which was statistically different when compared to that of the patients who had a worsened condition (0.3%) (*p* < 0.001), Table 2.

**Figure 2:**
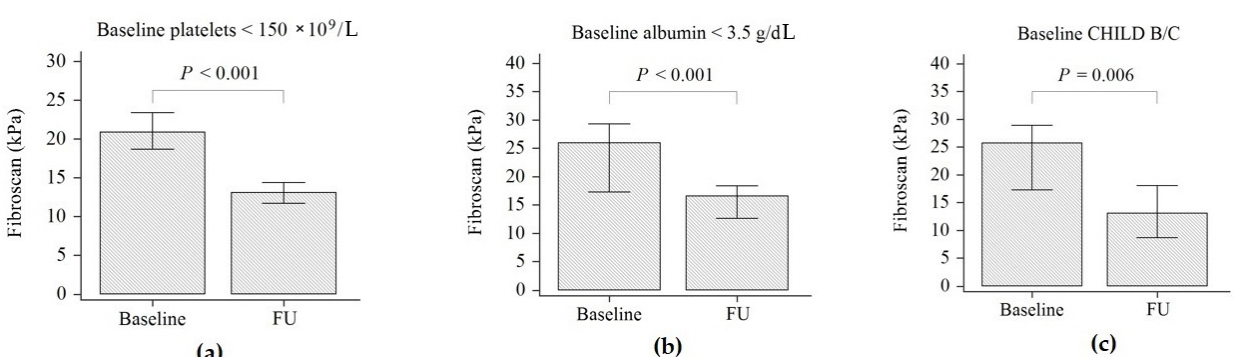
The liver stiffness changes during follow-up according to the baseline platelets count (**a**), albumin levels (**b**) and CHILD (**c**). status.

In the subgroup of patients with baseline platelets lower than 150 × 10^9^/L, the LS decreased by 1.6-fold (from 20.9 kPa to 13.0 kPa, *p* < 0.001, Figure 2a); similarly, in patients with baseline albumin levels lower than 3.5 g/dL, the LS decreased by 1.6-fold (from 26 kPa to 16.6 kPa, *p* < 0.001, Figure 2b). In patients with baseline CHILD B/C cirrhosis, the LS decreased during follow-up by 2-fold (from 25.7 kPa to 13.1 kPa, *p* = 0.006), Figure 2c.

## 4. Discussion

In this retrospective study of individuals with cured HCV-related cirrhosis, we showed that during the follow-up following viral eradication, a significantly higher proportion of patients had improved serum albumin levels as a biochemical marker of liver synthesis (66%). In addition, the overall median LS values improved from the baseline: 19.3 kPa vs. 11.6 kPa (*p* < 0.001). Conversely, the proportion of patients who showed improved indirect signs of portal hypertension (according to the Baveno criteria) was 19.4%, which was similar to that of patients who had a worsened condition. We then stratified our cohort according to the RESIST-HCV score, which has been strongly validated as a non-invasive tool to predict high-risk esophageal varices before and following antiviral treatment by DAAs. When applying the suggested cut-offs to identify the “low risk” group bearing high-risk varices, we found that a greater proportion of patients had shifted into this group (45.6% vs. 52.0%). This finding has a great relevance in this cohort, because it indirectly supports the beneficial impact of DAA treatment on portal hypertension, particularly with regard to high-risk varices, which are majorly prone to bleeding [12,13].

Despite liver cirrhosis being the last stage of the natural history of any chronic liver disease, it represents a still dynamic process that can progress toward more severe stages of decompensation and a rise in HCC. The use of DAAs in clinical practice is effective in curing HCV infection and has led to a global decline in its incidence, but the management of cirrhosis in clinical practice represents a major burden. Simple and readily available tools using clinical/biochemical markers are required to help physicians evaluate the progression of liver disease. From this perspective, albumin levels are a hallmark of liver synthesis, while platelet count is the earliest sign of subclinical portal hypertension and is included in the Baveno criteria for the management of portal hypertension. In our study, about 71% of patients classified as CHILD B/C moved to the CHILD A class (*p* < 0.001). The CHILD score is a strongly validated tool to evaluate liver function, and is commonly used to classify cirrhotic patients into decompensated (CHILD B/C) and compensated (CHILD A). Our results support the beneficial effect of viral eradication in cirrhotic patients, not only in terms of blocking the progression of liver disease, but also in improving synthesis and portal hypertension. In one study including 642 cirrhotic patients undergoing HCV eradication, about 29% of the total could achieve a significant improvement in liver function as measured by the MELD score, which is supportive for short-term prognosis [14]. In addition, the value of albumin levels as a marker for disease improvement in the cirrhotic population was retrospectively confirmed after the data extrapolation of four large trials conducted with DAAs [15], and these data were confirmed in another large multicenter study on either compensated and decompensated patients stratified by the CHILD score [16].

Interestingly, in the decompensated population with impaired liver synthesis, and in the subgroup with portal hypertension, there was a significant reduction in the LS values, ranging from 1.6 to 2-fold decrease. In clinical practice, LS represents an accurate and valuable tool to assess liver fibrosis indirectly and to rule in clinically significant portal hypertension (CSPH), according to the Baveno VII criteria [17]. The value of LS as a prognostic tool is not strongly validated yet, despite evidence supporting its role in the assessment of treatment response [7,18]. Our findings support the idea that LS can be used longitudinally to evaluate improvements in the cirrhotic population. Notably, the improvements in LS that occurred in this cohort did not reach a strong clinical significance because of the persistence of high values of stiffness (overall more than 10 kPa in the follow-up examination). However, these improvements may be considered from a mechanistic view. In fact, LS reflects the intrahepatic inflammation and parallel increasing fibrogenesis. The removal of the harmful factor alleviates liver inflammation, leading to lesser degrees of stiffness, even in cirrhotic livers, and may lead to lower rates of decompensation [19]. One study showed that among individuals with HCV-related cirrhosis undergoing viral eradication, post-treatment LS >20 kPa, but no pre-treatment LS values were independently associated with liver decompensation. This finding remarks on the usefulness of LS monitoring after the removal of the etiologic factor, and supports its role in risk stratification and prognostication [20].

The strength of this study is the large, single-center cohort of HCV-related cirrhosis that was retrospectively evaluated (almost 3000 individuals with successful SVR) and the follow-up data that could be extracted to analyze changes over time. However, some limitations need to be highlighted. The retrospective nature of the study led to insufficient data with regard to all parameters included in the CHILD score (bilirubin, presence of ascites or encephalopathy, INR). Similarly, the evaluation of concomitant harmful factors including alcohol intake could not be assessed. In particular, the absence of INR, another biochemical hallmark of liver synthesis, did not allow for comparisons with the albumin levels. With regard to portal hypertension, the main limitation was the absence of strong determinants for its diagnosis (HVPG, endoscopy, abdominal imaging), which was therefore based solely on platelet count. However, we believe that the evaluation of simple tools allows for quick, reliable assessments in the clinical setting, which was the aim of this analysis.

## 5. Conclusions

In conclusion, in a population of cirrhotic patients undergoing successful HCV eradication, follow-up evaluation showed an improvement in the LS and markers of liver synthesis and portal hypertension, suggesting the dynamicity of metabolic processes with potential improvements even in end-stage liver disease. Improvements in the LS, even in decompensated patients, suggest potential reductions in the decompensation rates following successful viral eradication.

## Figures and Tables

**Table 1:** The baseline clinical characteristics of the study cohort (N = 373).

Clinical Characteristics	Baseline	Follow-Up	*p*-Value
Age (years)	64 (57–77)	-	-
Male *n* (%)	267 (41.5)	-	-
Female *n* (%)	377 (58.5)	-	-
AST (IU/L)	69 (45–101)	23 (19–29)	<0.001
ALT (IU/L)	67 (43–111)	20 (16–27)	<0.001
GGT (IU/L)	66 (40–109)	24 (16–37)	<0.001
Albumin (g/dL)	4.1 (3.7–4.4)	4.4 (4.0–4.6)	<0.001
Platelet count × 10^9^/L	119 (86–158)	127 (90–170)	<0.001
“Low-risk” RESIST-HCV score *n* (%)	170 (45.6%)	194 (52%)	0.004
CHILD *n* (%)			
A	345 (94.4%)	357 (95.7%)	<0.001
B/C	28 (37.5%)	16 (4.3%)	
Liver stiffness (kPa)	19.3 (14.7–27)	11.6 (7.7–16.8)	<0.001

Footnote. ALT, alanine aminotransferases; AST, aspartate aminotransferases; GGT, gamma-glutamyl aminotransferases; HCV, hepatitis C virus.

**Table 2:** The proportion of patients who had improved or worsened indirect signs of portal hypertension and hepatic synthesis as described by the cut-offs for the platelet count and albumin levels (150 × 10^9^/L and 3.5 g/dL, respectively) Alb—albumin levels; PLT—platelet count.

	Baseline PLT < 150 (×10^9^/L)	Baseline PLT ≥ 150(×10^9^/L)		Baseline Alb < 3.5 (g/dL)	Baseline Alb ≥ 3.5 (g/dL)
**Follow-up** **PLT < 150 (×10^9^/L)**	204 (80.6%)	21 (17.5%)	**Follow-up** **Alb < 3.5 (g/dL)**	18 (34%)	1 (0.3%)
**Follow-up** **PLT ≥ 150 (×10^9^/L)**	49 (19.4%)	99 (82.5%)	**Follow-up** **Alb ≥ 3.5 (g/dL)**	35 (66%)	319 (99.7%)
Tot	253	120		53	320

## Data Availability

The data presented in this study are available on request from the corresponding author.

## Supplementary Materials

The following supporting information can be downloaded at: https://www.mdpi.com/article/10.3390/biology11081160/s1, Table S1: Direct Antiviral Agents (DDA) regimens administered to the study cohort.
