# Supplementary material for: Changes in Liver Stiffness and Markers of Liver Synthesis and Portal Hypertension following Hepatitis C Virus Eradication in Cirrhotic Individuals
Source: Biology (Basel). 2022 Aug 2;11(8):1160. doi: 10.3390/biology11081160 (PMC9404889; doi:10.3390/biology11081160)
Supplement: Supplementary file 1 [file biology-11-01160-s001.zip › biology-1820373-supplementary.pdf]

Supplementary Table S1. Direct Antiviral Agents (DDA) regimens administered to the study cohort.

**DAA regimens**	N (%)
Sofosbuvir/Ledipasvir (+/- Ribavirin)	200 (53.6%)
Sofosbuvir (+/- Ribavirin)	52 (13.9%)
Paritaprevir/Ritonavir/Ombitasvir + Dasabuvir (+/- Ribavirin)	42 (11.3%)
Sofosbuvir + Daclatasvir (+/- Ribavirin)	28 (7.5%)
Sofosbuvir/Velpatasvir (+/- Ribavirin)	22 (5.9%)
Sofosbuvir + Simeprevir (+/- Ribavirin)	15 (4.0%)
Paritaprevir/Ritonavir/Ombitasvir (+/- Ribavirin)	10 (2.7%)
Sofosbuvir + Velpatasvir + Voxilaprevir (+/- Ribavirin)	2 (0.5%)
Elbasvir/Grazoprevir (+/- Ribavirin)	1 (0.3%)
Glecaprevir + Pibrentasvir (+/- Ribavirin)	1 (0.3%)
